# Protective Effect of *Zizyphus lotus* L. (Desf.) Fruit against CCl_4_-Induced Acute Liver Injury in Rat

**DOI:** 10.1155/2019/6161593

**Published:** 2019-12-25

**Authors:** Noureddine Bencheikh, Mohamed Bouhrim, Loubna Kharchoufa, Mohammed Choukri, Mohamed Bnouham, Mostafa Elachouri

**Affiliations:** ^1^Laboratory of Physiology Genetic and Ethnopharmacology, URAC-40, Department of Biology, Faculty of Sciences, Mohammed First University, Oujda, Morocco; ^2^Faculty of Medicine and Pharmacy, Oujda, Morocco; ^3^Biochemistry Laboratory, Central Laboratory Service—CHU, Mohammed VI, Oujda, Morocco

## Abstract

In Morocco, “*Zizyphus lotus* L.” is one of the most widely and traditionally used plant species to treat various diseases, including liver disorders. The present study was conducted to evaluate the aqueous extract of *Zizyphus lotus* L. fruit against carbon tetrachloride- (CCL_4_-) induced liver damage in Wistar rats. The animals were daily treated with the aqueous extract of *Zizyphus lotus* L. fruit using two doses separately 200 and 400 mg/kg body weight for 14 days. CCL_4_ was injected intraperitoneally (1 ml/kg body weight) at two times, 7^th^ and 14^th^ days. At the end of the treatment, rats were sacrificed, and the blood was collected for the assessment of biochemical parameters. Moreover, the body weight as well as liver weight was determined. The injection of CCl_4_ to the rats induced various alterations such as the increase of relative liver weight, serum alkaline phosphatase, alanine aminotransferase, aspartate aminotransferase, total bilirubin, direct bilirubin, triglycerides, very low-density lipoproteins, total cholesterol (slight increase), creatinine, urea, uric acid, and malondialdehyde. On the contrary, the effects of CCL_4_ lead to the reduction in serum levels of high-density lipoprotein. However, the daily administration of the aqueous extract of *Zizyphus lotus* L. fruit to the injected rats with CCL_4_ restored this abnormal variation in these biochemical parameters to normal values. Based on the results obtained in this study, it seems that the aqueous extract of *Zizyphus lotus* L. fruit has an hepatoprotective effect against hepatic lesions induced by CCL_4_ in rats.

## 1. Introduction

The liver is one of the most important vital organs in our body; it takes care of several essential physiological functions such as metabolism, secretion, and storage [1]. Moreover, the liver plays a crucial role in the detoxification and excretion of endogenous and exogenous substances such as xenobiotics, pharmaceutical drugs, and viral infections [1, 2]. Thus, this indicated the vulnerability of the liver which is mostly threatened by the toxic effects of xenobiotic compounds, leading to hepatic insufficiency. In fact, the alteration of the liver is considered among the deadly diseases, and nearly 20,000 deaths and 250,000 new cases recorded each year are due to the liver disorder [3]. Furthermore, the treatment of the liver failure by conventional medicine could have adverse effects on the organism, especially on liver [4]. Hence, the alternative therapy remained an essential option, especially in the developing countries, such as Morocco, where the poverty and the precarious of sanitary system were dominated. In addition, in this country, traditional phytotherapy is well rooted [5]. Among the medicinal plants used by Moroccan people to treat various ailments, *Zizyphus lotus* L. (Desf) (ZLF) known in Morocco by its vernacular name “Sadra,” belonging to the family of *Rhamnaceae*, are widely distributed in arid and semiarid regions [6]. Several parts of ZLF have been used traditionally for the treatment of several conditions, such as liver disorders, urinary tract infections, gastrointestinal problems, insomnia, skin infections, diarrhea, and diabetes [7, 8]. Besides, this plant is well known by their safety [9]. Several pharmacological investigations of this species have been devoted to the treatment of various ailments such as antispasmodic [10], anti-inflammatory, analgesic [11], antiulcerogenic [12], gastroprotective [13], antidiabetic [14], and litholytic effects [7]. However, the hepatoprotective effects of the fruit of this plant species have not been investigated. Hence, in the present paper, we attempt to assess the hepatoprotective effect of the ZLF extract. Finally, we induced liver damage in the rat by intraperitoneal injection of carbon tetrachloride (CCl_4_), which is an excellent hepatotoxic compound most commonly used to induce liver toxicity in experimental rats [5]. In fact, this compound (CCl_4_), which is a colorless, odorless, volatile, and highly toxic, acts on the liver through the action of cytochrome P450 enzymes and later reacts with oxygen to form the highly reactive derivative trichloromethyl peroxyl radical inducing lipid peroxidation and damage the membranes of liver cells and organelles, leading to the swelling and necrosis of hepatocytes, and result in the release of cytosolic enzymes such as aspartate aminotransferase (AST), alanine aminotransferase (ALT), and alkaline phosphatase (ALP) into the circulating blood [15–17]. For these reasons, CCl_4_ has been widely used in animal models to investigate chemical toxin-induced liver damage [18, 19]. The intraperitoneal injection of CCl_4_ to the animals leads to the interaction of CCL_3_ with important cellular molecules that may be structurally or biologically functional such as proteins, lipids, and nucleic acids, causing liver damage and impaired liver function [20].

The aim of the current study is to evaluate the hepatoprotective effects of ZLF aqueous extract on the CCl_4_-induced acute liver toxicity in the rats.

## 2. Materials and Methods

### 2.1. Chemical Compounds

Aspartate aminotransferase (AST), alanine aminotransferase (ALT), alkaline phosphatase (ALP), total cholesterol (TC), triglycerides (TG), high-density lipoprotein (HDL-c), bilirubin direct and total, urea, creatinine, and uric acid (UA) kits were purchased from Biosystems, Spain. Silymarin, carbon tetrachloride (CCl_4_) was purchased from Sigma Chemicals, USA. All other reagents used in this study were of high quality and analytical grade.

### 2.2. Plant Material

The plant was collected from the Oujda region (Morocco) between September and October 2018. The identification of the plant species ZLF was performed by the expert botanist Mohammed Fennane from the scientific institute of the University Mohammed V. And, the specimen was deposited under the voucher number HUMPOM428 in the herbarium at University Mohammed I, Oujda (Morocco).

### 2.3. Preparation of the Plant Extract

The ZLF was grounded and transformed on powder. Then, the aqueous extract was prepared by the addition of 100 g of ZLF powder in 2000 mL of boiled distillated water, and the mixture was shaked for 20 minutes. The mixture was filtered, and the filtrate was evaporated in order to eliminate water and to obtain the extract in powder form.

### 2.4. Animals

Thirty Wistar rats (♂/♀ = 1) weighing between 180 and 270 g, and thirty-six Swiss albino mice weighing between 20 and 30 g were used in this study. The animals were kept in the plastic cages with free access to food and water and were adapted to standard pet shop conditions (23°C, 12 h dark/12 h bright) for 15 days before treatment. All rats were cared in compliance with the internationally accepted Guide for the Care and Use of Laboratory Animals, published by the US National Institutes of Health (NIH publication no. 85-23, revised in 1985).

### 2.5. Acute Toxicity Study

The acute oral toxicity study was conducted in accordance with OECD Guidelines (425) [21]. Administration of the aqueous extract of ZLF from 200 mg to 2000 mg/kg body weight showed no signs of toxicity. From 1/10 to 1/5 of the upper limit dose (2000 mg/kg) was chosen as the level of examination to evaluate the hepatoprotective effect of the aqueous extract of ZLF.

### 2.6. Experimental Protocol and Procedure

After 15 days of acclimatization, rats were divided into five groups (*n* = 6; ♂/♀ = 1) [22] and treated as follows: a normal control group received only the distilled water (10 mL/kg), a negative control group injected by CCL_4_, a positive group treated with a hepatoprotective drug (silymarin) at a dose of 40 mg/kg, and two treated groups received the doses 200 and 400 mg/kg of the aqueous ZLF extract. suitable product by the oral administration for 14 days. In addition, all treated groups except the control group were previously injected intraperitoneally by the CCl_4_ solubilized in olive oil (1/4; v/v) at a dose of 1 mL/kg at two times, the 7^th^ and 14^th^ days of treatment. Body weight of the rats was measured before and after the treatment.

### 2.7. Blood Sampling

Twelve hours after the last dose of CCl_4_ injection, all rats were anesthetized by light ethyl ether inhalation and sacrificed. Blood samples were collected from the carotid arteries and centrifuged at 3000 rpm for 10 min, at 4°C to separate the plasma. The separated plasma was stored at −20°C for further assessments. In addition, the liver was weighed and conserved for the preparation of the liver homogenate (10% w/v) in sodium phosphate buffer (pH 7.0) and stored at −20°C to evaluate the level of malondialdehyde (MDA).

### 2.8. Measurement of Serum Biochemical Parameters

Aspartate aminotransferase and alanine aminotransferase were evaluated using the IFCC method [23]. The paranitrophenyl phosphate method was used to determine alkaline phosphatase [24], glycerol phosphate oxidase method to determine triglycerides [25], diazonium salt method to determine bilirubin total [26], ultra-HDL assay to determine high-density lipoprotein, enzymatic method to determine total cholesterol [27], diazo reaction method to determine bilirubin direct [26], uricase-peroxidase method to determine uric acid [28], urease-GLDH method to determine urea [29], and kinetic alkaline picrate method to determine creatinine [30]. For each parameter, the repetition of three times was done using commercial reagent kits.

The low-density lipoprotein level was estimated according to [31] the following formula:

(1) $$\begin{array}{c} \text{LDL}‐\text{c}=\text{TC}-\left(\text{HDL}‐\text{c}+\text{VLDL}‐\text{c}\right). \end{array}$$

The following formula VLDL-c = TG/5 [32] was applied to infer the level of very low-density lipoprotein.

### 2.9. Determination of Malondialdehyde

Lipid peroxidation in the liver was evaluated by the assay of MDA according to the method of Buege and Aust [32]. This method consists of quantifying the level of TBARS production. After having prepared the homogenate of the liver, 0.5 mL of this homogenate was added to 0.5 mL of trichloroacetic acid (30% w/v) and then subjected to centrifugation for 10 minutes (3500 rpm) in 4°C. Then, 1 mL of the supernatant obtained after centrifugation was homogenized with 1 mL of thiobarbituric acid (0.67% w/v) and placed in boiled water at 100°C for 10 minutes and then immersed in ice to stop the reaction. The optical density of the test mixture was measured spectrophotometrically at a wavelength of 535 nm. The results were expressed in moles of MDA produced per gram of tissue at 37°C, using the following molar extinction coefficient:

(2) $$\begin{array}{c} 1.56\times 105\;{\text{M}}^{-1}\cdot {\text{cm}}^{-1}. \end{array}$$

### 2.10. Statistical Analyses

The results are expressed as the mean ± SEM. The statistical difference between the negative control group and the treated groups was calculated by analysis of variance (ANOVA) using GraphPad Prism 5 software, San Diego, CA, USA. The difference was considered significant if *P* < 0.05, moderately significant if *P* < 0.01, and highly significant if *P* < 0.001.

## 3. Results

### 3.1. Acute Toxicity Study

The administration of the aqueous extract of ZLF does not represent any mortality or behavioral changes of the animals at the dose limit of 2000 mg/kg body weight. Doses 1/10 and 1/5 of the dose limit were chosen to assess the hepatoprotective effect of the aqueous extract of ZLF.

### 3.2. Effect of ZLF on the Relative Liver Weight and Body Weight Gain

**Figure 1:**
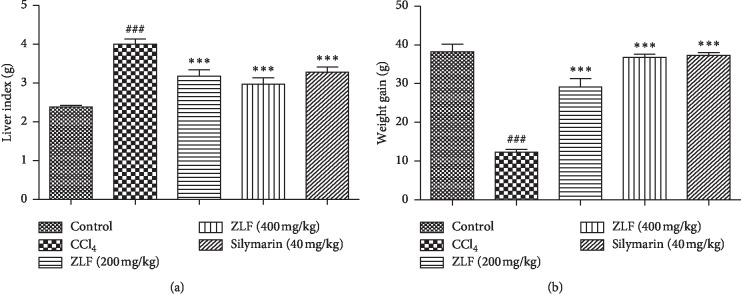
Effects of the ZLF aqueous extract on growth parameters in CC1_4_ -poisoned rats. Data are mean ± SEM, *n*=6. ^###^*P* < 0.001 related to the control group. ^*∗∗∗*^*P* < 0.001 versus the CCl_4_ group.

The body weight and relative liver weight of the animals in all study groups are presented in Figure 1. A significant reduction in body weight was observed along with increase in the liver weight relative to the group (control). However, the groups treated with 200 and 400 mg/kg ZLF, respectively, underwent significantly attenuated hepatoxicity (*P* < 0.001) in reversal of deleterious effect of CCl_4_ injection, with a pronounced dose response. The administration of 400 mg/kg of ZLF extract results in protective effects on the weight of liver and body comparable with that of the silymarin reference drug.

### 3.3. Effect of ZLF on AST, ALT, and ALP

**Figure 2:**
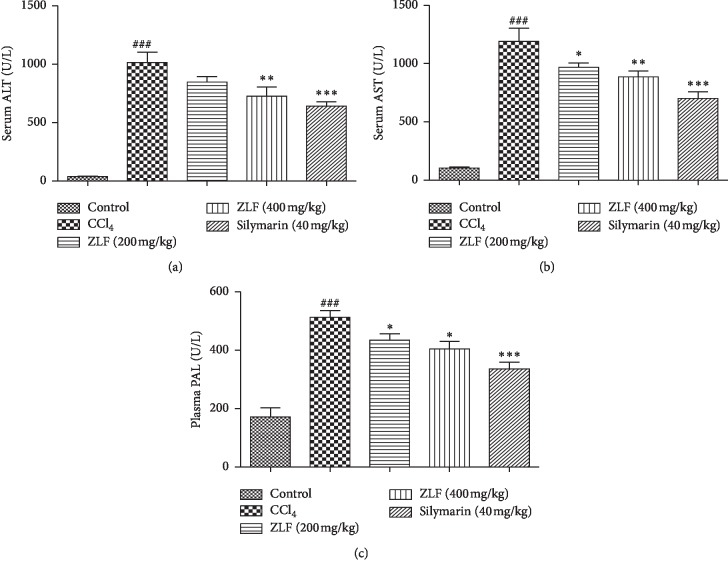
Effects of the ZLF aqueous extract on CCl_4_ induced the alterations in plasma hepatic markers. (a) ALT: alanine aminotransferase, (b) AST: aspartate aminotransferase, and (c) PAL: alkaline phosphatase. Data are mean ± SEM, *n*=6. ^###^*P* < 0.001 related to the control group. ^*∗*^*P* < 0.05, ^*∗∗*^*P* < 0.01, and ^*∗∗∗*^*P* < 0.001 versus the CCl_4_ group.

The effect of ZLF aqueous extract at two doses (200 and 400 mg/kg) on plasma liver markers (AST, ALT, and ALP) after induction of liver injury by intraperitoneal injection of CCl_4_ is shown in Figure 2. The injection of CCl_4_ to the rats provoked a significant increase (*P* < 0.001) in the plasma levels of ALT, AST, and ALP compared with the rats of the control group. However, the administration of the aqueous extract of ZLF at two different doses (200 and 400 mg/kg) showed a significant dose-related reduction in the plasma levels of liver markers (ALT, AST, and ALP) compared with the CCl_4_ group.

The effect of the aqueous extract of ZLF at a dose of 400 mg/kg on ALT and AST levels tends towards bioequivalence with silymarin treatment, a potent drug commonly administered against liver fibrosis and considered standard of care in that respect.

### 3.4. Effect of ZLF on Direct and Total Plasma Bilirubin

**Figure 3:**
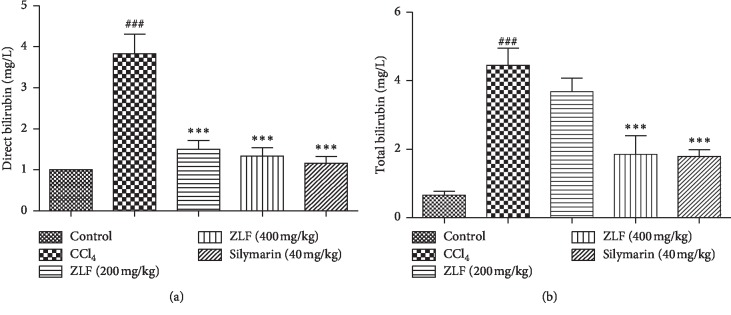
Effects of the ZLF aqueous extract on plasma direct bilirubin (a) and total bilirubin (b) in CCl_4_-intoxicated rats. Values are mean ± SEM, *n*=6. ^###^*P* < 0.01 related to the control group. ^*∗∗∗*^*P* < 0.001 related to the CCl_4_ control group.

The plasma levels of direct and total bilirubin in all animals in the study groups after treatment with the aqueous extract of ZLF are shown in Figure 3. A significant (*P* < 0.001) increase in the levels of direct and total bilirubin was observed in rats exposed to CCl_4_ compared with the control group. In addition, this increase indicates that CCl_4_ causes the dysfunction of hepatic excretory cells. However, the treatment of animals with ZLF aqueous extract at doses 200 and 400 mg/kg induced a significant (*P* < 0.001) dose-dependent decrease in plasma bilirubin concentrations (direct and total) compared with rats in the group exposed to CCl_4_. The effect of dose administration (400 mg/kg) is comparable with that of the silymarin drug (40 mg/kg).

### 3.5. Effect of ZLF on Plasma Total Cholesterol and Triglycerides

**Figure 4:**
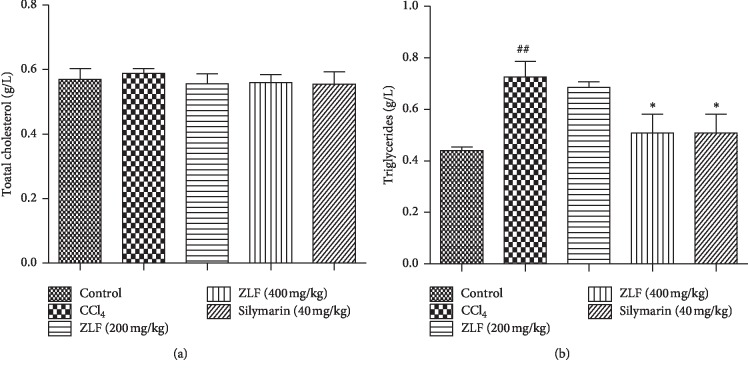
Effects of the ZLF aqueous extract on plasma total cholesterol (a) and triglycerides (b) in CCl_4_-intoxicated rats. Data are mean ± SEM, *n*=6. ^##^*P* < 0.01 compared with the normal control group. ^*∗*^*P* < 0.05 related to the CCl_4_ control group.

Plasma concentrations of total cholesterol and triglycerides were investigated in this study to evaluate the effect of aqueous extract of ZLF on liver metabolic function (Figure 4). In this study, a significant (*P* < 0.05) elevation of triglyceride levels was observed in the animals group exposed to CCl_4,_ compared with the control group, but no change was shown on the total cholesterol level. However, a significant (*P* < 0.05) dose-dependent reversal of triglyceride levels after daily oral intake of ZLF extract was seen in the treatment groups. In addition, a similar effect on triglyceride reduction was observed for the treated groups 400 mg/kg and the silymarin drug (40 mg/kg).

### 3.6. LDL-c, VLDL-c, and HDL-c

**Figure 5:**
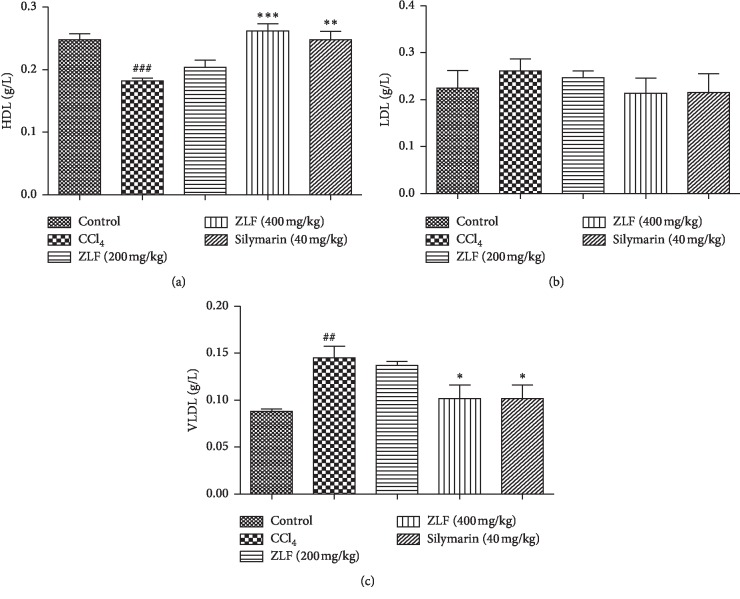
Effects of the aqueous extract of ZLF on plasma HDL-c (a), LDL-c, (b) and VLDL-c (c) in CCl_4_ intoxicated rats. Data are mean ± SEM, *n*=6. ^###^*P* < 0.001, ^##^*P* < 0.01 related to control group. ^*∗*^*P* < 0.05, ^*∗∗*^*P* < 0.01, ^*∗∗∗*^*P* < 0.001 versus CCl_4_ group.

The effect of the ZLF aqueous extract at 200 and 400 mg/kg on plasma lipoprotein levels was evaluated after exposure of rats to CCL_4_ (Figure 5). The group of animals injured by CCL_4_, a significant (*P* < 0.01) increase in the amount of VLDL, a significant (*P* < 0.001) reduction in HDL levels, and no change in the LDL content were observed, compared with the group control rats. However, the imbalance in the HDL and VLDL levels was slightly corrected by the administration of ZLF aqueous extract at doses 200 and 400 mL/kg. The efficacy of the dose 400 mL/kg on VLDL quantity recovery is quite similar to that of silymarin and almost comparable in maintaining HDL levels.

### 3.7. Effect of ZLF on Plasma Uric Acid, Urea, and Creatinine

**Figure 6:**
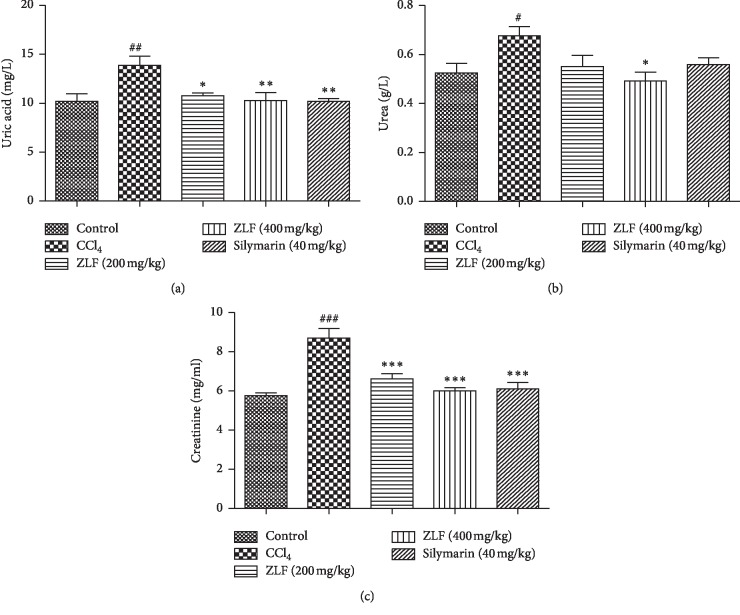
Effects of aqueous extract of ZLF on plasma uric acid (a), urea (b), and creatinine (c) in CCl_4_-intoxicated rats. Data are mean ± SEM, *n*=6. ^###^*P* < 0.001, ^##^*P* < 0.01, and ^#^*P* < 0.05 versus the control group. ^*∗*^*P* < 0.05, ^*∗∗*^*P* < 0.01, and ^*∗∗∗*^*P* < 0.001 versus the CCl_4_ control group.

The nephroprotective effect of aqueous extract of ZLF was evaluated by measuring plasma levels of renal function biomarkers such as creatinine, uric acid, and urea (Figure 6). The group injured by the intraperitoneal injection of CCl_4_ showed a significant increase in the creatinine (*P* < 0.001), uric acid (*P* < 0.01), and urea (*P* < 0.05) concentrations in the plasma. In addition, the silymarin-treated group at the dose of 40 mg/kg resulted a significant decrease in measured parameters. However, the daily intake of ZLF aqueous extract at the doses 200 and 400 ml/kg induced a highly significant decrease in creatinine (*P* < 0.001) (*P* < 0.001) and uric acid ((*P* < 0.05) (*P* < 0.01)), respectively, and a slight reduction in urea level. In addition, the ZLF aqueous extract showed a dose-dependent nephroprotective effect against CCl_4_-induced liver toxicity.

### 3.8. Effect of ZLF on MDA Level

**Figure 7:**
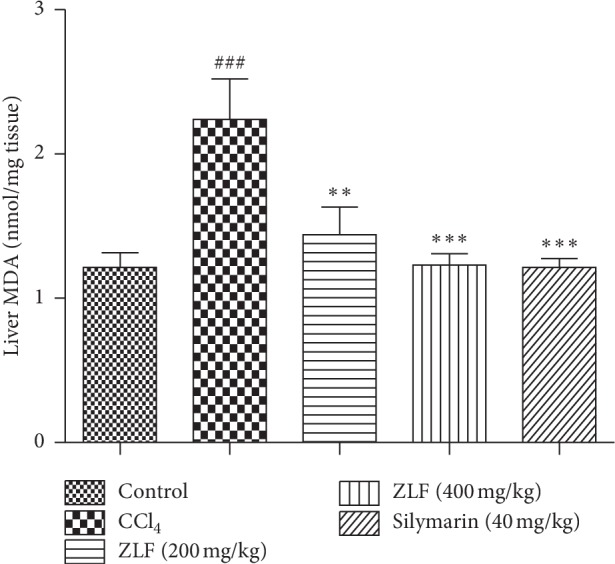
Effects of aqueous extract of ZLF on lipid peroxidation in CCl_4_-intoxicated rats. Values are mean ± SEM, *n*=6. ^###^*P* < 0.01 related to the normal control group. ^*∗∗*^*P* < 0.01 and ^*∗∗∗*^*P* < 0.001 versus the CCl_4_ control group.

As shown in Figure 7, a significant (*P* < 0.001) increase in lipid peroxidation in the CCl_4_ group was observed, as indicated by high level of MDA compared with the healthy group. The doses 200 and 400 mg/kg of ZLF extract showed a significant (*P* < 0.01) (*P* < 0.001) reduction in the level of MDA. The reduction in the MDA level at 400 mg/kg was nearly identical to the standard of care, silymarin.

## 4. Discussion

In the current study, carbon tetrachloride (CCl_4_) was used to induce the liver toxicity to assess the hepatoprotective effect of the ZLF aqueous extract. CCl_4_ appears in the body as a xenobiotic, and by the action of the cytochrome P_450_ oxygenase system, the liver transforms the carbon tetrachloride into two free radicals; trichloromethyl peroxyl and trichloromethyl [4]. However, these two free radicals are highly reactive, acting on the microsomal membrane of the liver through their binding on proteins or on polyunsaturated fatty acids of the endoplasmic reticulum [33, 34]. In addition, the fixing of these free radicals to unsaturated lipids in the presence of oxygen causes lipid peroxidation [34]. The latter leads to the deterioration of enzymatic and metabolic functions of the liver, such as the synthesis of albumins proteins, and inhibits the storage of sugar and cholesterol in liver cells [35]. It is well known that liver prevention against CCl_4_ is based either on stimulating the regeneration of liver cells or on the system of antioxidants that protect the liver against CCl_4_-induced acute toxicity by trapping free radicals formed from it and oxidizing during the process [36]. For this purpose, several chemical compounds derived from plants that have a powerful antioxidant effect such as polyphenol, flavonoids, coumarins, and alkaloids were recognized for the prevention against liver toxicity [37–40].

In this study, the hepatoprotective effect of ZLF aqueous extracts was evaluated on the hepatic toxicity induced in rats by the intraperitoneal injection of CCl_4_. The assessment of liver damage caused by CCl_4_ was based on measurements of plasma levels of markers enzymes (ALT, AST, and ALP), total cholesterol, triglycerides, VLDL, LDL, HDL, MDA, and bilirubin (total and direct). In addition to hepatic markers, we also evaluated the markers of renal excretory function by measuring plasma levels of urea, uric acid, and creatinine. Injection of CCl_4_ to the rat induced a significant increase in the plasma levels of ALT, AST, ALP, bilirubin (total and direct), urea, uric acid, creatinine, triglycerides, VLDL, LDL, and MDA, and a significant reduction in HDL level compared with the control group. After the administration of the aqueous extract of ZLF at two doses (200 and 400 mg/kg), reversal of the above changes occurred compared with the CCl_4_ group. These results indicate that the aqueous extract of ZLF has potential to protect from liver injury causing the abnormal variation in the plasma biochemical parameters caused by CCl_4_ intraperitoneal injection. It seems that the hepatoprotective effect of ZLF on liver damage, caused by CC1_4_, may be due to the antioxidant activity of the ZLF extract. In addition, one study demonstrated that the aqueous extract of ZLF is rich in polyphenols and flavonoids [7]. These chemical compounds are known to have antioxidant properties by trapping free radicals produced by liver involvement by CCl_4_ [41]. It can be concluded that ZLF has a hepatoprotective effect by reduction of the free radicals caused by the injection of CCl_4_.

## 5. Conclusion

Based on our findings, we conclude that the aqueous extract of *Zizyphus lotus* L. fruit has a potent hepatoprotective effect against CCl_4_-induced liver injury in rats. Thus, the study provides experimental evidence and clearly justifies traditional claims and their use in the treatment of liver diseases. Based on the safety, the use valued in traditional medicine of the ZLF and the current promising results, we pretend, in the future, to investigate, some studies on this plant in the aim to find the bioequivalence between the dose 400 mg/kg of the ZLF'extract and 40 mg/kg of silymarin reference drug.

## Data Availability

No data were used to support this study.
